# Macroparasite Fauna of Alien Grey Squirrels (*Sciurus carolinensis*): Composition, Variability and Implications for Native Species

**DOI:** 10.1371/journal.pone.0088002

**Published:** 2014-02-05

**Authors:** Claudia Romeo, Lucas A. Wauters, Nicola Ferrari, Paolo Lanfranchi, Adriano Martinoli, Benoît Pisanu, Damiano G. Preatoni, Nicola Saino

**Affiliations:** 1 Department of Biosciences, Università degli Studi di Milano, Milan, Italy; 2 Department of Theoretical and Applied Sciences, Università degli Studi dell'Insubria, Varese, Italy; 3 Department of Veterinary Sciences and Public Health, Università degli Studi di Milano, Milan, Italy; 4 Department of Ecology and Biodiversity Management, Muséum National d’Histoire Naturelle, Paris, France; Università degli Studi di Napoli Federico II, Italy

## Abstract

Introduced hosts populations may benefit of an "enemy release" through impoverishment of parasite communities made of both few imported species and few acquired local ones. Moreover, closely related competing native hosts can be affected by acquiring introduced taxa (spillover) and by increased transmission risk of native parasites (spillback). We determined the macroparasite fauna of invasive grey squirrels (*Sciurus carolinensis*) in Italy to detect any diversity loss, introduction of novel parasites or acquisition of local ones, and analysed variation in parasite burdens to identify factors that may increase transmission risk for native red squirrels (*S. vulgaris*). Based on 277 grey squirrels sampled from 7 populations characterised by different time scales in introduction events, we identified 7 gastro-intestinal helminths and 4 parasite arthropods. Parasite richness is lower than in grey squirrel's native range and independent from introduction time lags. The most common parasites are Nearctic nematodes *Strongyloides robustus* (prevalence: 56.6%) and *Trichostrongylus calcaratus* (6.5%), red squirrel flea *Ceratophyllus sciurorum* (26.0%) and Holarctic sucking louse *Neohaematopinus sciuri* (17.7%). All other parasites are European or cosmopolitan species with prevalence below 5%. *S. robustus* abundance is positively affected by host density and body mass, *C. sciurorum* abundance increases with host density and varies with seasons. Overall, we show that grey squirrels in Italy may benefit of an enemy release, and both spillback and spillover processes towards native red squirrels may occur.

## Introduction

Biological invasions are one of the major causes for biodiversity loss worldwide [1]–[4], therefore the attention on mechanisms and processes driving alien species settlement, their spread and their subsequent impact on native ecosystems is constantly growing. In recent years several authors recognised that micro- and macroparasites can play an important role in biological invasions, affecting alien species settlement and mediating their interaction with native species (reviewed in: [5]–[7]). It has been observed in different taxa of both plants and animals, that invasive species often loose part of their parasite fauna during the introduction process and that, in most cases, this reduction in parasite species is not completely compensated by the acquisition of local parasites from the new environment [8],[9]. Consequently, in a new settlement area, alien species are usually less heavily parasitised (both in terms of richness and prevalence) than in their native range. This release from natural “enemies” may increase individual viability and population growth rate, facilitating invaders' settlement and spread ([10]–[13], but see [14]).

Additionally, parasites that are successfully introduced by invaders may potentially become a major threat for native species and greatly affect the outcome of interspecific competition [15]. If spillover (i.e. transmission of infectious agents from reservoir populations to sympatric animals, [16]) between introduced and susceptible local hosts occurs, the parasite impact can indeed be very severe, since native species can be maladapted to alien parasites (e.g. [17]). Moreover, invaders may also successfully acquire local parasites, altering their epidemiology and increasing the abundance of infective stages in the environment (via the increased density of competent hosts). This acquisition may in turn exacerbate the impact of local parasites on native hosts (spill-back hypothesis, [18]) and also lead to an increase in health risks for humans [19]. On the contrary, if invaders are infected by local parasites but are not competent hosts, the alien species may act as a sink and lessen the parasite impact on native species (dilution effect, [20]–[22]).

One of the most cited examples of parasites playing a role in biological invasions, is the Squirrelpoxvirus (SQPV) mediating the competition between introduced North American Eastern grey squirrels (*Sciurus carolinensis*) and native Eurasian red squirrels (*Sciurus vulgaris*) in Great Britain and Ireland [23], [24]. The alien species, introduced in Great Britain at the end of the 19th century, acts as healthy carrier for the SQPV, whereas the virus, in most cases, is lethal for red squirrels [25]. As a result, in sites where SQPV is present, the replacement of the native species by grey squirrels is much accelerated than when only food exploitation competition occurs [23].

During the second half of last century the grey squirrel was repeatedly introduced also to Northern Italy, becoming a threat to red squirrels in continental Europe [26], [27]. In all the sites where the alien species is present, the red squirrel disappeared or is declining because of interspecific competition for resources which reduces female reproductive success and juvenile recruitment [28]–[31].

Despite the attention received by SQPV, there is a generalised lack of information about the potential role played by macroparasites in the competition between these two species. One of the reasons for this is that the effects of macroparasites are generally sublethal and more difficult to detect: most of the emerging infectious diseases reported for wildlife are caused by microparasites since they are more likely to produce massive mortality events associated with clearly recognisable symptoms [32]. Still, macroparasites can have a great impact on host population dynamics [33] and affect interspecific competition to the point of causing the exclusion of one host over the other, as shown for example by [34] between ring-necked pheasant and grey partridge in the U.K.

Hereafter, we investigate the composition of the macroparasite fauna of the grey squirrel in Northern Italy to inquire if this alien species introduced exotic parasites to Europe, or acquired local parasites (i.e., if there are any premises for parasite spillover and/or spill-back towards native species). We also compare macroparasite richness of grey squirrels introduced to Italy to what is known in the literature about their native range. We expect that richness in Italy will be lower than in North America, the more so in recently established populations (i.e. founded during the last two decades) compared to “older” populations (i.e. founded in 1948).

Finally, in order to point out potential extrinsic and intrinsic factors that may increase parasite transmission to red squirrels, we explored variation in burdens of dominant parasite taxa infesting grey squirrels according to season, host density, sex and body mass.

## Materials and Methods

A total of 277 grey squirrel individuals were sampled between 2011 and 2013 in 7 study areas located in Northern Italy, four in Piedmont region and three in Lombardy. In all Piedmont sites, red squirrels went extinct between 1992 and 2000 and all the study areas are included in the metapopulation which originated from the release of 4 squirrels in 1948 [35]. On the contrary, Lombardy populations originated from independent releases that took place during the last 20 years [26] and red squirrels still persist at low numbers in some sites or are present with small populations nearby (Romeo et al., unpublished data). The number of founders of Lombardy nuclei is unclear, but each population was likely founded by few (<10) individuals (Romeo et al., unpublished data). Sampling was carried out specifically for scientific research on parasites and within a European Community LIFE Project (LIFE09 NAT/IT/00095 EC-SQUARE) aimed at controlling or eradicating Italian grey squirrel populations. Squirrels were captured using Tomahawk live-traps (model 202, Tomahawk Live Trap Co., Wisconsin, USA) and immediately euthanised by CO_2_ inhalation, following EC and AVMA guidelines [36]–[38] and with authorizations by Lombardy Region, Cuneo Province and Italian Institute for Environmental Protection and Research (ISPRA). In each sampling area, at least three trapping sessions (minimum 3 continuous days each) were carried out in different seasons. Traps were baited with a mixture of walnuts, hazelnuts and corn and, depending on day lenght, were checked two to three times a day to avoid animals from being overly stressed. For each individual we recorded sex, age class (juveniles or adults, based on weight and reproductive conditions), reproductive condition and body mass to the nearest gram. Each carcass was immediately placed in a sealed plastic bag and stored at −20°C for later examination.

Two hundred and fourteen grey squirrels were examined for both ectoparasites and gastro-intestinal helminths, 17 only for ectoparasites and 46 only for helminths. In the laboratory, defrozen carcasses were first combed on a white surface using a flea comb, to collect ectoparasites. Arthropods (fleas, ticks and sucking lice) were counted and stored in ethanol 70% for later identification. To search for helminths, the whole gastro-intestine from oesophagus to rectum was removed. Each tract (stomach, small intestine, caecum and colon-rectum) was dissected separately, washed with tap water and its content filtered through two sieves (lumen 0.40 and 0.03 mm, respectively). The content of each tract was then examined separately under a stereo-microscope (10× magnification) and helminths were counted and stored in lactophenol or ethanol 90% for identification. Morphological identification of both arthropods and helminths was carried out using a microscope equipped with camera lucida and was based on [39] for the genus *Trichuris* and [40] for the family Hymenolepididae. For details on the identification of the other taxa, see [41].

### Statistical Analysis

To assess whether our sampling effort was adequate, we compared observed richness of both helminths and ectoparasites with estimated richness computed using EstimateS software (Version 9, R. K. Colwell, http://purl.oclc.org/estimates). The software estimates species richness extrapolating the asymptote of species accumulation curves (i.e. a plot of cumulative species richness against sampling effort) at each level of sampling effort. To avoid biases due to the order in which samples are drawn from the data set, the program averages richness estimates over many randomized runs (in our case, 100 runs). The program produces also several non-parametric estimators that add to the species richness an estimate based on the abundance of rare species. As suggested by [42], we chose the mean values of Chao2 estimator as it is the estimator that performs best with parasite distributions.

We explored the effect of host-linked factors (sex and body mass) and extrinsic factors (capture season and density of hosts) on the abundance (no. of parasites/host) of the most prevalent helminth and ectoparasite. Definition of seasons was based on temporal changes in tree squirrel behaviour and food availability, as described in previous studies (winter, December-February; spring, March-May; autumn, September-November, e.g. [43], [44]). 15 individuals trapped in summer were excluded from the analysis to avoid problems with small sample size. To obtain density of hosts in each study site, we first estimated the population size using a catch-effort depletion model, assuming variable trapping effort, according to [45], as implemented in the R (R Core Team, http://www.r-project.org) package \texttt{fishmethods} [46]. This model improves standard linear regression methods to estimate the number of individuals present at the start of a series of trapping sessions (Y-variable), based on the number of animals trapped and removed (X-variable) in subsequent sessions, assuming a closed population during the entire trapping period [47]. We are confident about the assumption of closed populations sincee population size was estimated at the start of removal sampling, using sufficiently short trapping periods that did not include the autumn, the major period of dispersal in this species [48]. Furthermore, all our trapping sites are high-quality mixed broadleaf forest fragments far-between each other, surrounded by a low-quality matrix (i.e. cultivated land). Hence they are spatially distinct and partly-isolated from other sites with grey squirrels. Study sites were then classified according to relative density, calculated as population sizes divided by trapping areas, as follows: low-density sites (host density <3 squirrels/ha), medium-density sites (3< host density<7) and high-density sites (host density>7). Categories were set following available literature on grey squirrel population dynamics in different habitat types (e.g. [48], [49]). Since all sampling sites had similar habitat conditions (i.e. mixed deciduous woods, low elevation, similar weather conditions), no other environmental variables were considered. Before the analysis, we examined all the explanatory variables for covariance and no major collinearity issues leading to statistical confounding effects were detected. The parasites considered in statistical analyses showed an aggregated distribution in the host population [50], thus variation in their abundance was analysed using Generalised Linear Models (GLMs) with negative binomial error distribution and log link-function. We first fitted full models with all fixed effects and their second order interaction and then obtained minimum models through backward elimination of non-significant factors. Interpretation of final models was based on pair-wise *t*-tests of Differences of Least Square Means (DLSM), applying sequential Bonferroni correction [51] for multiple comparisons.

Unless otherwise specified, all values and parameter estimates are reported as mean (±SE).

GLMs were performed using SAS/STAT 9.2 software (Copyright © 2009, SAS Institute Inc., Cary, NC, USA).

### Ethics Statement

All sampling protocols were chosen to minimise animal stress and suffering. Traps were checked two to three times a day, depending on day length and handling time minimised to prevent animals from being overly stressed. Method of euthanasia (CO_2_ inhalation) was chosen and performed according to what stated in AVMA guidelines [38] and also followed the guidelines determined by the EEC in Directives 86/609/EEC and 93/119/EEC and further developed in [36], [37]. Euthanasia was carried out immediately on the field to avoid transportation and extended captivity of captured animals. Permits for trapping and culling grey squirrels were granted by Italian Institute for Environmental Protection and Research (ISPRA), Lombardy Region (Authorization No.: 3892, 02/05/2011) and Cuneo Province (Permit No.: 473, 12/05/2011).

## Results

### Parasite Fauna Composition

In 260 grey squirrels examined we identified a total of 6 gastro-intestinal nematode species and one cestode species (Table 1), with a resulting estimated richness of 7.0±0.4 SD species (Chao2 estimator). In addition, ten unidentified nematode specimens (7 larvae and 3 adult females) and one unidentified oxyurids were found in 11 different grey squirrels. No acantocephalan or trematode species were found. Individual richness ranged from 0 to 4 with a mean value of 0.8 species/host. The most abundant helminth was the nematode *Strongyloides robustus* with a total prevalence of 56.6% and a mean intensity (mI) of 16.9±2.1 worms infested/host. All the other identified nematodes were rare, with prevalence below 7% and most of them also with low intensities of infestation (Table 1). *Trichostrongylus calcaratus* was found in 17 hosts (6.5%, mI = 1.9±0.3), whereas 1 and 26 adult specimen of *T. retortaeformis* were found in 2 squirrels (0.8%). Adult males and immature females of *Trichuris muris* were found in 11 hosts (4.2%, mI = 1.3±0.2). In 4 grey squirrels (1.5%) we found respectively 1, 2, 2 and 4 specimens of *Aonchotheca annulosa*, and in 6 hosts (2.3%) we found the oxyurid *Trypanoxyuris sciuri* with intensities ranging from 1 to 379 worms. Finally, in one individual (0.4%) we found a single cestode specimen belonging to the family Hymenolepididae.

**Table 1:** Helminth species infecting grey squirrels in Piedmont and Lombardy populations.

Helminth species	Piedmont		Lombardy		Total	
Host age	n (p)	mI ± SE	n (p)	mI ± SE	n (p)	mI ± SE
**Juvenile**	**N = 19**		**N = 14**		**N = 33**	
*Strongyloides robustus*	11 (58%)	6.7±2.4	6 (43%)	7.7±3.1	17 (52%)	7.1±1.9
*Trichuris muris*	3 (16%)	*1; 1; 1*	0	–	3 (9%)	*1; 1; 1*
*Trypanoxyuris* (*R.*) *sciuri*	0	–	1 (7%)	*1*	1 (3%)	*1*
**Adult ♀**	**N = 59**		**N = 54**		**N = 113**	
*Strongyloides robustus*	39 (66%)	15.4±3.5	30 (56%)	14.2±4.3	69 (61%)	14.9±2.7
*Trichostrongylus calcaratus*	12 (20%)	2.2±0.4	1 (2%)	*1*	13 (12%)	2.1±0.3
*Trichuris muris*	6 (10%)	1.5±0.3	0	–	6 (5%)	1.5±0.3
*Aonchotheca annulosa*	3 (5%)	*2; 2; 4*	1 (2%)	*1*	4 (4%)	*1; 2; 2; 4*
*Trypanoxyuris* (*R.*) *sciuri*	0	*–*	1 (2%)	*6*	1 (1%)	*6*
*Trichostrongylus retortaeformis*	0	–	1 (2%)	*26*	1 (1%)	*26*
Strongylida [gen. sp.]	3 (5%)	*1; 1; 1*	1 (2%)	*1*	4 (4%)	*1; 1; 1; 1*
Oxyurida [gen. sp.]	0	–	1 (2%)	*1*	1 (1%)	*1*
**Adult ♂**	**N = 63**		**N = 51**		**N = 114**	
*Strongyloides robustus*	33 (52%)	24.3±5.4	28 (55%)	19.3±5.8	61 (54%)	22.0±3.9
*Trichostrongylus calcaratus*	4 (6%)	*1; 1; 1; 2*	0	–	4 (4%)	*1; 1; 1; 2*
*Trichuris muris*	2 (3%)	*1; 1*	0	*–*	2 (2%)	*1; 1*
*Trypanoxyuris* (*R.*) *sciuri*	1 (2%)	*1*	3 (6%)	*1; 13; 379*	4 (4%)	*1; 1; 13; 379*
*Trichostrongylus retortaeformis*	0	*–*	1 (2%)	*1*	1 (1%)	*1*
Hymenolepididae [gen. sp.]	1 (2%)	*1*	0	*–*	1 (1%)	*1*
Strongylida [gen. sp.]	4 (6%)	*1; 1; 1; 1*	2 (4%)	*1; 1*	6 (5%)	1±0

N: number of host examined; n: number of infected hosts; p: prevalence; mI: mean intensity (no. parasites infected/hosts; when number of infected hosts <5, worm counts in italic).

Excluding the single cestode specimen, richness in the two regions was consistent, with 5 nematodes each, since *T. muris* and *T. retortaeformis* were found only in Piedmont and Lombardy, respectively (see Table 1).

A total of 4 ectoparasite arthropod species was found on 231 grey squirrels: two fleas, one sucking louse and one ixodid tick (Table 2). The corresponding estimated richness was 4.0±0.5 SD species (Chao2 estimator). Individual richness ranged from 0 to 3, with a mean value of 0.5 species/host. The most prevalent species, found on 60 hosts (26.0%, mI = 2.7±0.3 parasites infested/host), was the flea *Ceratophyllus sciurorum*. The other most common arthropod was the sucking louse *Neohaemathopinus sciuri* that was found on 41 hosts (17.7%; mI = 3.6±0.8). Finally, 4 specimens of the tick *Ixodes acuminatus* were found on 4 squirrels (1.7%) and a single specimen of the flea *Ctenocephalides felis* was found on one squirrel (0.4%). With the exception of the single specimen of *C. felis*, the other three ectoparasite species were found both in Piedmont and Lombardy (see Table 2).

**Table 2:** Arthropod species infesting grey squirrels in Piedmont and Lombardy populations.

Arthropodspecies	Piedmont		Lombardy		Total	
Host age	n (p)	mI ± SE	n (p)	mI ± SE	n (p)	mI ± SE
**Juvenile**	**N = 17**		**N = 12**		**N = 29**	
*Neohaemathopinus* *sciuri*	7 (41%)	3.7±0.9	0	–	7 (24%)	3.7±0.9
*Ceratophyllus* *sciurorum*	6 (35%)	3.2±1.3	1 (8%)	*3*	7 (24%)	3.1±1.1
**Adult ♀**	**N = 62**		**N = 44**		**N = 106**	
*Neohaemathopinus* *sciuri*	13 (21%)	2.4±0.5	2 (4%)	*2; 10*	15 (14%)	3.0±0.7
*Ceratophyllus* *sciurorum*	16 (26%)	2.5±0.4	6 (14%)	3.0±1.4	22 (21%)	2.6±0.5
*Ctenocephalides* *felis felis*	1 (2%)	*1*	0	*–*	1 (1%)	*1*
*Ixodes* *acuminatus*	0	–	1 (2%)	*1*	1 (1%)	*1*
**Adult ♂**	**N = 54**		**N = 42**		**N = 96**	
*Neohaemathopinus* *sciuri*	16 (30%)	4.1±1.7	3 (7%)	*1; 2; 3*	19 (20%)	3.9±1.5
*Ceratophyllus* *sciurorum*	22 (41%)	2.4±0.5	9 (21%)	3.0±0.5	31 (32%)	2.6±0.4
*Ixodes* *acuminatus*	1 (2%)	*1*	2 (5%)	*1; 1*	3 (3%)	*1; 1; 1*

N: number of host examined; n: number of infested hosts; p: prevalence; mI: mean intensity (no. parasites infested/hosts; when number of infested hosts <5, worm counts in italic).

### Factors Affecting Parasite Infection

The most common helminth and arthropod infecting grey squirrels and thereby considered for abundance analysis were, respectively, *S. robustus* and *C. sciurorum*.

**Figure 1:**
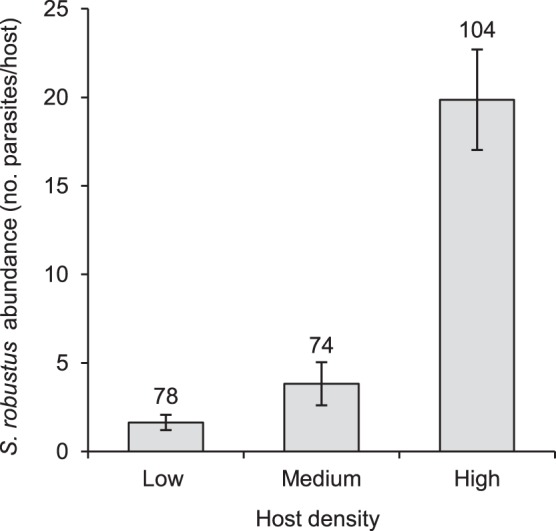
Variation of *S. robustus* abundance by host density. Mean abundance of *S. robustus* (sample size above standard error bars) varied with density of hosts in the site (p<0.0001). Squirrels living in high-density sites were more infested than individuals living in medium- and low-density sites (both sequential Bonferroni adjusted p<0.0001) and squirrels living in medium-density sites were more infested than in low-density sites (adjusted p = 0.0008).

**Figure 2:**
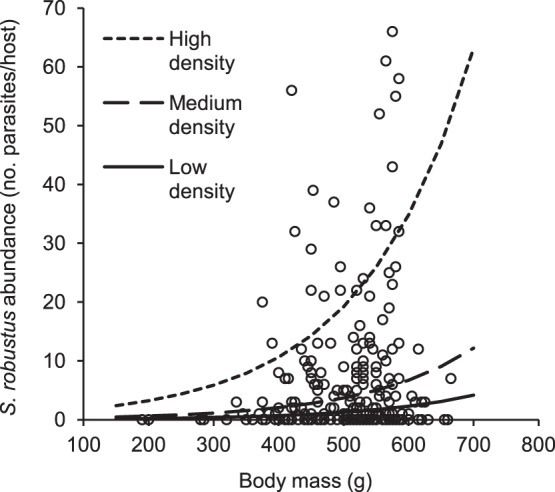
Variation of *S. robustus* abundance by host body mass. Relationship between *S. robustus* abundance and host body mass: observed values (blank circles) and values predicted by the model at different host densities (lines). Host body mass had a positive effect on *S. robustus* abundance (p = 0.0005; parameter estimate: 0.0059±0.0017 SE).

*S. robustus* abundance (number of worms/host) varied with density of hosts in the study site and host body mass (Table 3). Squirrels living in high-density sites were more infested than individuals living in medium- and low-density sites (both adjusted p<0.0001, Figure 1) and squirrels living in medium-density sites were more infested than in low-density sites (adjusted p = 0.0008). Host body mass had a positive effect on *S. robustus* abundance (p = 0.0005, Figure 2).

**Table 3:** Minimum selected model of the effects of host characteristics and environmental variables on parasite abundance (no. of parasites/host).

Dependent variable	Source of variation	?^2^	df	P	Parameter estimate (±SE)
***S. robustus*** ** abundance**	Host density	95.3	2	<0.0001	
	Body mass	12.2	1	0.0005	0.0059±0.0017
***C. sciurorum*** ** abundance**	Host density	18.5	2	<0.0001	
	Season	39.4	2	<0.0001	

**Figure 3:**
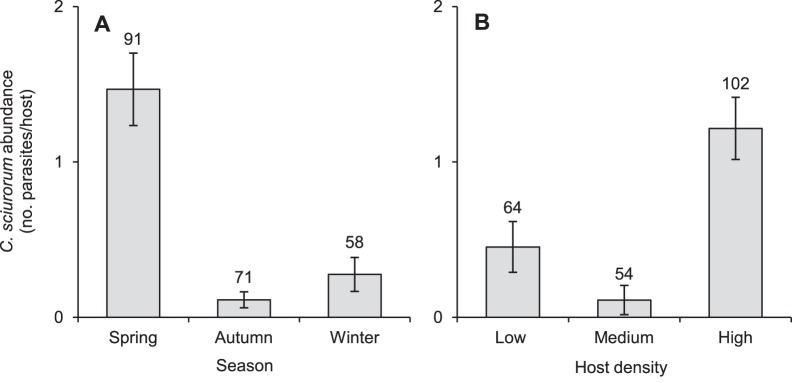
Variation of *C. sciurorum* abundance by season (A) and host density (B). Mean abundance of *C. sciurorum* (sample size above standard error bars) varied during different seasons (p<0.0001) and at different host densities (p<0.0001). Squirrels trapped in spring were more infested than in autumn and winter (both sequential Bonferroni adjusted p<0.0001) and animals living in high-density sites were more infested then those living in medium- and low-density populations (both adjusted p<0.008).

*C. sciurorum* abundance varied with season and density of hosts in the site (Table 3). Squirrels trapped in spring were more infested than in autumn and winter (both adjusted p<0.0001, Figure 3A), and animals living in high-density sites were more infested then those living in medium- and low-density populations (both adjusted p<0.008, Figure 3B).

## Discussion

The parasite fauna of grey squirrels introduced to Northern Italy is poor, with 7 gastro-intestinal helminth species and 4 ectoparasite arthropod species. Observed richness of both helminth and ectoparasite species is consistent with richness computed using Chao2 estimator, indicating that the low number of species encountered is not a result of inadequate sampling effort.

The most abundant helminth is *S. robustus*, a North American nematode common in grey squirrels and other Nearctic sciurids in their native range (e.g. [52]–[54]). Also *T. calcaratus* is a Nearctic parasite commonly found in several squirrel species, but its primary host is the North American cotton-tail rabbit (*Sylvilagus floridanus*, [55]). Indeed, in Italy *T. calcaratus* is found only in areas were the introduced cotton-tail rabbit is present, suggesting that the lagomorph likely acts as primary host of this nematode. Both these North American helminths had already been reported in Europe: *T. calcaratus* in alien cotton-tail rabbits [56] and *S. robustus* in a few Italian red squirrels co-inhabiting with grey squirrels [41]. All the other nematodes we found are Eurasian species: *T. muris* and *T. retortaeformis* are common parasites of wood mice (*Apodemus* spp., [39]) and wild rabbits (*Oryctolagus cuniculus*, [57]) respectively, *A. annulosa* is a generalist nematode infesting a wide variety of mammals [58], including squirrels (e.g. [59], [60]) and *T. sciuri* is the dominant helminth of Eurasian red squirrels [41], [61]. *T. sciuri* was found only in few individuals and mostly in sites were red squirrels are still present or were present until a few years ago, suggesting that this parasite may not adapt well to grey squirrels and probably needs the presence of its native host to successfully persist. The most common ectoparasite species infesting grey squirrels in Italy are the flea *C. sciurorum* and the sucking louse *N. sciuri*. The first is the main flea species of Eurasian red squirrels [41], [62], whereas *N. sciuri* is a species with a Holarctic distribution, commonly found both on the Eurasian red squirrel and on North American tree squirrels [63]. Interestingly, opposite to *T. sciuri*, *C. sciurorum* was found in all the sites, even in areas where the red squirrel went extinct decades ago, indicating that the flea got adapted to the new host and can complete its cycle without the native species being present. The other recorded arthropods are rare: the cosmopolitan flea *C. f. felis*, whose primary host is the domestic cat, and the Palearctic tick *I. acuminatus*, reported also rarely on red squirrels [41].

Only two helminths (*S. robustus* and *T. calcaratus*) and two arthropods (*C. sciurorum* and *N. sciuri*) have prevalence above 5% and only three of these species (*S. robustus*, *C. sciurorum* and *N. sciuri*) are present in all the sampled populations. All the other species are found only locally and are likely linked to the presence of other primary hosts. More important, contrary to our expectations, parasite species richness is consistent in Piedmont and Lombardy and does not seem to be affected by populations' origin or “age”.

According to the available literature (excluding studies with sample size below 50 hosts), grey squirrels in their native range are parasitised by at least 8 gastro-intestinal helminth and 7 ectoparasite species (mites excluded) with prevalence above 5% (Table 4). Thus, compared to our results (we found only 2 helminths and 2 ectoparasites above the same prevalence), parasite richness reported for grey squirrels in their native range is higher, both for gastro-intestinal helminths and ectoparasites. This holds true even limiting the comparison to a smaller spatial scale, to studies carried out in the North Eastern part of the United States (i.e. the likely native range of the animals introduced to Italy): [64] and [65] reported respectively 4 helminths and 5 helminths and 5 arthropods infecting grey squirrels with prevalence above 5%. Moreover, we found only three of the species reported in North America (*S. robustus*, *T. calcaratus* and *N. sciuri*, the latter having a Holarctic distribution), whereas several parasites common in the Nearctic region are completely missing in Italy (e.g. the nematode *Citellinema bifurcatum* or the flea *Orchopeas howardii*). It is also interesting to notice that the parasite fauna of grey squirrels introduced to the U.K. is different than in Italy: for example in the U.K. the flea *O. howardii*, absent in Italy, is commonly observed (e.g. [66]), on the contrary, the nematode *S. robustus*, the most abundant helminth in Northern Italy, has never been reported.

**Table 4:** Most prevalent gastro-intestinal helminths and arthropods (excluding mites) parasitizing grey squirrels in North America.

Parasitespecies	Prevalence	Samplesize	Reference
**Gastro-intestinal helminths**			
*Strongyloides robustus*	28%–86%	62–270	[64], [65], [81]
*Citellinema bifurcatum*	35%–45%	62–270	[65], [81], [82]
*Bohmiella wilsoni*	14%–29%	175–270	[65], [81]
*Heligmodendrium hassalli*	7%–92%	53–270	[52], [64], [65], [81], [82]
*Capillaria americana*	7%–14%	62–270	[64], [65], [81]
*Trichostrongylus calcaratus*	4%–16%	175–270	[65], [81]
*Syphacia thompsoni*	5%	175–270	[65], [81]
*Enterobius sciuri* *(T. bicristata?)*	2%–26%	175–270	[64], [65], [81]
**Arthropods**			
*Neohaematopinus sciuri*	33%–81%	53–106	[52], [65], [83]
*Hopopleura sciuricola*	32%–55%	53–106	[52], [65], [83]
*Enderleinellus longiceps*	2%–68%	67–106	[65], [83]
*Orchopeas howardii*	51%–76%	53–106	[52], [65], [83]
*Amblyomma americanum*	22.4%–32.8%	67	[83], [84]
*Ixodes scapularis*	1.5%–47.8%	67	[83], [84]
*Dermacentor variabilis*	4.5–8.9	67	[83], [84]

Only parasites that were recorded by more than one author and with maximum prevalence >5% are reported. Studies with sample size <50 hosts were excluded.

Abundance of both the main helminth, *S. robustus*, and the main ectoparasite, *C. sciurorum*, in Italian grey squirrel populations varied with density of grey squirrels in the study sites. Abundance of both parasites was significantly higher in squirrels living in high-density populations. This result is not surprising since positive density dependence in parasite transmission is expected from theoretical studies [67] and a positive relationship between host density and abundance has indeed been observed in several taxa [68]–[70]. This pattern could also explain why *C. sciurorum* abundance varied also with seasons and was higher in spring than in autumn or winter. The peak in infestations levels occurs after the first breeding period of grey squirrels [48], when population density and contact among individuals increase and presence of potential hosts for fleas is higher. On the contrary, [41] reported an abundance peak of *C. sciurorum* in Eurasian red squirrels in autumn, after their second reproduction. Red squirrels are known to delay or even skip spring reproduction in different forest types, and often reach maximum population density in autumn rather than in spring [71], [72]. Hence, this difference in seasonal abundance of the flea between the two hosts could be particularly alarming since, in areas where the two species co-inhabit, the normal seasonal distribution of *C. sciurorum* could be altered by the presence of grey squirrels, with an increased risk of transmission on red squirrels during spring. Furthermore, *S. robustus* abundance varied positively with host body mass. This result may be partly due to age growth [73], but may also be a consequence of the fact that larger animals offer a wider skin surface for *S. robustus* larvae to penetrate [68]. Moreover, in many tree squirrel species, body mass is positively correlated with dominance rank and/or home range size (e.g. [74]–[76]). Thus, parasite abundance may be related to individual boldness: having larger home ranges and engaging more in explorative and mating behaviour, larger, dominant squirrels may have higher exposure to infestation [77].

Hence, first of all, our findings demonstrate that grey squirrels introduced to Italy lost part of their original parasite fauna (even several dominant species), and although they acquired some Palearctic parasites, their number does not compensate the number of species lost. This holds true even when we compare our data only with studies carried out in a small portion of grey squirrel's native range and exclude parasite species that were only reported by a single author, making our conclusion conservative. Since all Italian grey squirrel populations were founded by a small number of individuals (i.e. had a low “propagule pressure”, [78]), it is likely that some parasite species never reached the new range due to stochastic founder effects or were lost during the initial stages of invasion due to low host-densities insufficient for their transmission and persistence [79]. To test whether grey squirrels actually benefit from this parasite loss (i.e. whether the enemy release hypothesis holds true) further research is needed. Our results also suggest that *S. robustus* was introduced to Italy with the grey squirrel and that red squirrels likely acquired it by spillover from the alien species [41]. It should be noted that *S. robustus* is also suspected to mediate the competition between two species of North-American flying squirrels (*Glaucomys* spp.: [80]). We also show that the opposite process occurs: grey squirrels acquired the flea *C. sciurorum* and, to a lesser extent, the oxyurid nematode *T. sciuri* from red squirrels. Examining whether the acquisition of these parasites by the grey squirrels is altering their epidemiology with repercussions for red squirrels and investigating the consequences of *S. robustus* spillover for the native species are both priorities and specific aims of ongoing research.
